# Doxorubicin metabolism moderately attributes to putative toxicity in prodigiosin/doxorubicin synergism in vitro cells

**DOI:** 10.1007/s11010-020-03864-x

**Published:** 2020-08-04

**Authors:** Shian-Ren Lin, Chun-Shu Lin, Ching-Cheng Chen, Feng-Jen Tseng, Tsung-Jui Wu, Lebin Weng, Ching-Feng Weng

**Affiliations:** 1 Graduate Institute of Cancer Biology and Drug Discovery, Taipei Medical University, Taipei, 110 Taiwan; 2 Department of Radiation Oncology, Tri-Service General Hospital, National Defense Medical Center, Taipei, Taiwan; 3 Camillian Saint Mary’s Hospital Luodong, Luodong, 26546 Yilan Taiwan; 4 Department of Orthopedics, Hualien Armed Force General Hospital, Hualien, 97144 Taiwan; 5 Functional Physiology Section, Department of Basic Medical Science, Xiamen Medical College, Xiamen, 361023 China; 6 Institute of Respiratory Disease, Department of Basic Medical Science, Xiamen Medical College, Xiamen, 361023 China

**Keywords:** Doxorubicin, Prodigiosin, Drug synergism, Drug metabolism, Cardiotoxicity

## Abstract

Doxorubicin (Dox) is a widely neoplasm chemotherapeutic drug with high incidences of cardiotoxicity. Prodigiosin (PG), a red bacterial pigment from *Serratia marcescens*, has been demonstrated to potentiate Dox’s cytotoxicity against oral squamous cell carcinoma cells through elevating Dox influx and identified as a Dox enhancer via PG-induced autophagy; however, toxicity of normal cell remains unclear. This study is conducted to evaluate putative cytotoxicity features of PG/Dox synergism in the liver, kidney, and heart cells and further elucidate whether PG augmented Dox’s effect via modulating Dox metabolism in normal cells. Murine hepatocytes FL83B, cardio-myoblast h9c2, and human kidney epithelial cells HK-2 were sequentially treated with PG and Dox by measuring cell viability, cell death characteristics, oxidative stress, Dox flux, and Dox metabolism. PG could slightly significant increase Dox cytotoxicity in all tested normal cells whose toxic alteration was less than that of oral squamous carcinoma cells. The augmentation of Dox cytotoxicity might be attributed to the increase of Dox-mediated ROS accumulation that might cause slight reduction of Dox influx and reduction of Dox metabolism. It was noteworthy to notice that sustained cytotoxicity appeared in normal cells after PG and Dox were removed. Taken together, moderately metabolic reduction of Dox might be ascribed to the mechanism of increase Dox cytotoxicity in PG-induced normal cells; nevertheless, the determination of PG/Dox dose with sustained cytotoxicity in normal cells needs to be comprehensively considered.

## Introduction

Rendering non-tissue specific characteristics, doxorubicin (Dox) has wide indications, e.g., leukemia, neuroblastoma, breast, and ovarian carcinoma, and most recurrent or metastatic cancer in cancer regimen [1], and it also has severe adverse effects in cancer patients. 31–51% of Dox is excreted via biliary tract in their original form and < 10% of Dox metabolite is eliminated via urine after intake [2, 3]. Three pathways of Dox metabolism are under investigation: one-electron reduction, two-electron reduction, and deglycosylation, which carried about 50% of Dox out of cells (remaining 50% will be excreted from their original form) [4]. One-electron reduction occurs in mitochondria which operates by NADH-ubiquinone oxidoreductase (NDUFS), xanthine oxidase (XOD), and a cytosolic enzyme NADPH: quinone oxidoreductase (NQO). The end-product of one-electron reduction is Dox-semiquinone, whose ROS activity is the main cause of oxidative stress. Two-electron reduction pathways which aldo/keto reductase (AKR) and carbonyl reductase (CBR) are the main metabolic pathway of Dox detoxification. Dox-deglycosylation is a minor route, which produces Dox-aglycone by NQO [5–7]. Along with the trigger mechanism, the cytotoxicity of Dox is considered to be via two approaches: apoptotic activation, which refers to hematopoietic inhibition, mucositis, and enteritis; and oxidative stress which causes most hepatotoxicity, nephrotoxicity, and cardiotoxicity [8, 9].

In clinic, Dox-induced cardiotoxicity is the most divergent of concerned toxicity. The incidence of Dox-induced cardiomyopathy is positively correlated to cumulative dose, which increased from 4% (500–550 mg/m^2^) to 36% (> 600 mg/m^2^) [10]. To minimize the adverse effect of Dox and keeping its anticancer efficacy, nanocarriers have been developed and some researchers have focused on dissolving these obstacles via liposomal-coated Dox (Lipo-Dox). However, the cytotoxicity of liposomal Dox is 10 times less than that of free-form Dox, which might increase the risk of chemoresistance induced [11]. The lower cytotoxicity of Lipo-Dox than free form of Dox sheds light on the possibility of adjuvant involvement in promoting Dox’s antitumor efficacy without raising its normal cell toxicity.

In cancer treatment, the term “adjuvant therapy” is referred to the regimen based on the synergistic effect or combination of two or more therapeutic drugs, usually a lower-dose therapy followed by a primary therapy for reducing cancer recurrence [12]. Clinically, adjuvant therapies for cancer treatment are usually combining surgery with chemotherapy or radiotherapy for efficaciously potentiating the effects of two treatments or chemicals, and whether this efficacy is indicative of a positive or negative response [13]. Generally, it is common to combine two current chemotherapeutic agents in one regimen, *i.e.*, Dox with tamoxifen, paclitaxel plus Dox, and cyclophosphamide or carboplatin with docetaxel [14]. However, clinical trials revealed the effects of polymorphism for adjuvant chemotherapy and the biomarkers for efficacy estimation were not completely or totally defined [15–18]. Accordingly, additional agents for enhancing the efficacy of adjuvant chemotherapeutic drugs or extending patient polymorphism need to developed using this new strategy. Previously, Dox accumulation caused by prodigiosin (PG)-priming has been confirmed in OSCC through the elevation of Dox influx [19]. However, the caused damage of PG/Dox combination in known organs, e.g., liver, kidney, and heart were not assessed. In this study, putative hepato-, nephro-, and cardiotoxicity of PG/Dox combinations and the underlying regulation are explored using an in vitro cellular assay.

## Material and methods

### Chemicals

Chemicals were obtained from various sources: purified PG was from Dr. Yu-Hsin Chen (National Museum of Marine Biology and Aquarium, Pingtung, Taiwan), liposome-coated Dox nanoparticle (Lipo-Dox) was provided by TTY Biopharm Company Limited (Taipei, Taiwan), general chemicals from Sigma-Aldrich (Merck KGaA, Darmstadt, Germany), medium and reagents for cell culture from Thermo-Fisher (Waltham, MA, USA), and inhibitors, antibiotics for selection, and lentiviral activation particle from Santa Cruz Biotechnology (Dallas, TX, USA) in this study. All reagents for cell culture were dissolved in dimethyl sulfoxide as a 1000X working stock and stored under dark, − 20 °C environment except lentiviral activation particle (at − 80 °C) and antibiotics for selection (4 °C).

### Cell culture

A total of 3 cell lines were from different sources: murine normal hepatocyte FL83B and cardio-myoblast h9c2 from Bioresource Collection and Research Center (BCRC, Hsinchu, Taiwan), and human renal cortex/proximal tubule epithelial cell HK-2 from Prof. Yaw-Syan Fu (Department of Biomedical Science and Environment Biology, Kaohsiung Medical University, Kaohsiung, Taiwan) were applied to test the hypothesis in this study. Three cell lines were cultured in different mediums and were all supplied with 10% fetal bovine serum and 1% antibiotic/antimycotic and 0.2% normocin™: FL83B in Kaighn-modified Ham’s F12 medium (F12K), h9c2 in Dulbecco’s modified Eagle medium (DMEM), and HK-2 in DMEM/F12 with additional 0.5 ng/mL epidermal growth factor (EGF). The cultural condition was set as 37 °C, 5% CO_2_, and saturated humidity. Culture medium was renewed once every 2 days. As cell confluence reached 80%, cells were detached by 0.25% Trypsin/EDTA for experiments. Cell passage was kept within 20 passages for controlling the uniformity of experiments.

### Cytotoxicity assay

Cytotoxicity analysis was performed by MTT (3-(4,5-dimethylthiazol-2-yl)-2,5-diphenyltetrazolium bromide) staining with viable cells as described in a previous study [19]. Briefly, 1 × 10^4^ cells/well of normal cells were inoculated into 96-well plate and incubated under cultural conditions overnight. Then, cells were treated with 0.5 μM of PG for 12 h followed by 2.5 μM of Dox treatment for 12 h. Afterward, 1 mg/mL MTT solution (in PBS) was added to the wells (final concentration 0.5 mg/mL) and incubated for 4 h. Finally, cultural medium was replaced with 50 μL of dimethyl sulfoxide and the optical density (OD) at 570 nm was measured by a Multiskan™ FC microplate photometer (Thermo-Fisher). Cytotoxicity was calculated from the OD ratio between untreated (control as 1) and treated groups.

### Cell cycle analysis

Cell cycle of treated cells was measured by propidium iodide (PI)-staining and flow cytometry according to the literature [19]. 1 × 10^6^ cells/well of normal cells were seeded into 6-well plate. Cells were treated with PG/Dox as described in the previous section. After treatment, cells and supernatant from each well was collected individually by 0.25% trypsin/EDTA, washed by pre-warm PBS, and fixed by ice-cold 70% ethanol/PBS under − 20 °C at least overnight. Fixed cells were washed out ethanol by PBS and stained with 20 μg/mL of propidium iodide coupling with 0.1% Triton X-100 and 0.2 mg/mL RNase A for 1 h at 37 °C. Fluorescent intensity from PI in each cell was determined by Cytomics™ FC500 flow cytometer (Beckman-Coulter, Fullerton, CA, USA). 1 × 10^4^ data from each well were collected and plotted histogram by flow cytometry software. The gating of each cell cycle state was performed according to the histogram from untreated controls.

### Dox influx assay

A Dox influx assay was performed by the accumulation of Dox-transporter substrate rhodamine 123 (R123) in the literature [19]. 1 × 10^4^ cells/well of normal cells were inoculated into a 96-well plate. Then, the cells were incubated with 0.5 μM PG for 12 h and 2 μM R123 at cultural conditions for 1 h. The cells were washed by pre-warm PBS and lysed by 0.1% triton X-100 for 10 min and fluorescent intensity at 485/538 nm was determined by Alphascreen™ microplate reader (Perkin-Elmer, Waltham, MA, USA). Dox influx was represented by fluorescent percentage from untreated (control) and treated groups.

### Gene overexpression

Lentiviral-mediated gene overexpression was performed by Lentiviral activation particle, which followed the protocol provided by the manufacturer (https://datasheets.scbt.com/protocols/CRISPR_Lenti_Activation_Protocol.pdf). 1.5 × 10^5^ cells/well of each cell were inoculated into 12-well plate and incubated in culture conditions overnight for cell attachment. 10 μL of lentivirus particle and 1.5 μL of 10 mg/mL polybrene were mixed in 1 mL of culture medium (with 2% FBS), added to each cell, and incubated in culture conditions overnight. After lentiviral infection, transfected cells were selected by incubation with 350 μg/mL hygromycin S for 8–24 h. Transfection was re-confirmed by measurement of cytotoxicity after treating with selected antibiotics for 24 h. Transfected cells were used to measure cytotoxicity change of PG/Dox compared with un-transfected cells and desired protein expression using Western blotting.

### Statistical analysis

Collected data were plotted with GraphPad Prism V7.04 (La Jolla, CA, USA) and performed analysis of variance (ANOVA) coupled with Dunnett’s test or Tukey’s range test as Post hoc comparison. Data were shown by mean ± SD from at least 3 independent experiments. As data were significantly different (*p* < 0.05) from untreated control or Dox alone, column of the data would be marked “*” or “#”, respectively.

## Results

### Cytotoxicity of PG/Dox synergism against normal cells

**Fig. 1:**
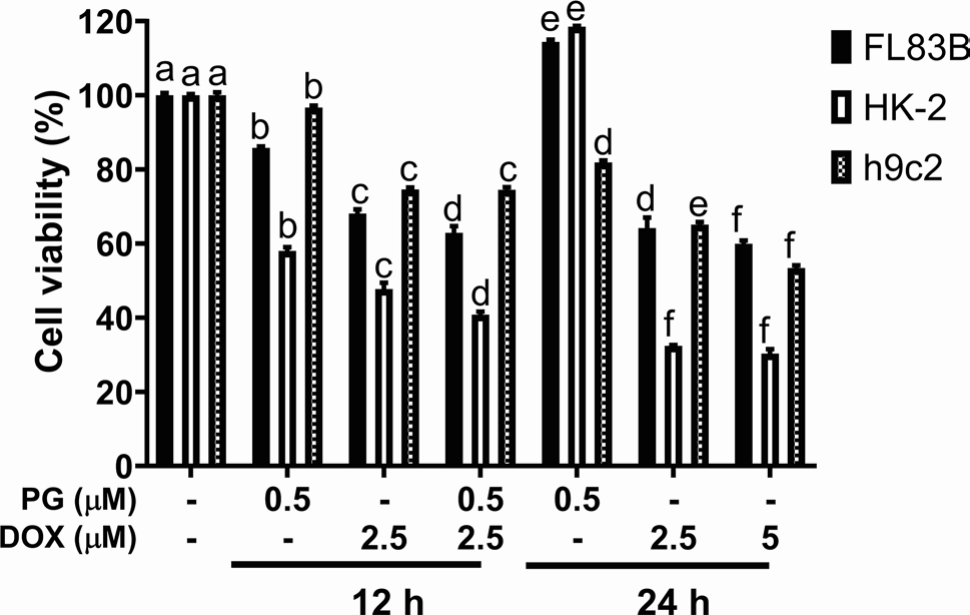
Cytotoxicity of PG/Dox against normal cells. FL83B, HK-2, and h9c2 cells were sequentially treated with PG/Dox for 12 and 24 h, respectively, and cell viability was measured by MTT assay. Data were represented with mean ± SD from four independent experiments. Columns significantly different letter (*p* < *0.05*) to each other would be labeled with different letters

To access putative toxicity from PG/Dox synergism in liver, kidney, and heart, FL83B (murine normal hepatocyte), HK-2 (human kidney proximal tubule epithelial cell), and h9c2 (murine normal cardio-myoblast) were sequentially treated with PG and Dox for 12 h, respectively. Comparing with PG or Dox alone treatment for 12 h, the cytotoxicity of PG/Dox exhibited significantly increase in 62.9 ± 1.8% (FL83B), 40.8 ± 0.8% (HK-2), and 74.5 ± 0.7% (h9c2), respectively, whose cell viabilities after Dox treatment were 68.1 ± 1.1% (FL83B), 47.7 ± 1.7% (HK-2), and 74.6 ± 0.5% (h9c2), respectively (Fig. 1). This result indicated cytotoxic enhancement also appeared in normal cells. However, the cell viability of PG/Dox in normal cells was higher trend (no significance) when compared with 2.5 μM Dox treating for 24 h. When the concentration of Dox alone increased to 5 μM, cell viability of three different cells dropped to 59.9 ± 0.9% (FL83B), 30.3 ± 1.2% (HK-2), and 53.4 ± 0.7% (h9c2), respectively. There were all significantly lower than that of PG/Dox treatment (Fig. 1). The results indicated the enhancement of Dox cytotoxicity combined with PG-priming; however, its cytotoxic enhancement was not higher than that of Dox alone treated with 24 h. Moreover, the range of cytotoxic enhancement in normal cells was smaller than OSCC [19], which could reinforce the potential of PG/Dox combination for clinical use.

### Death property of PG/Dox-induced cell death in normal cells

**Fig. 2:**
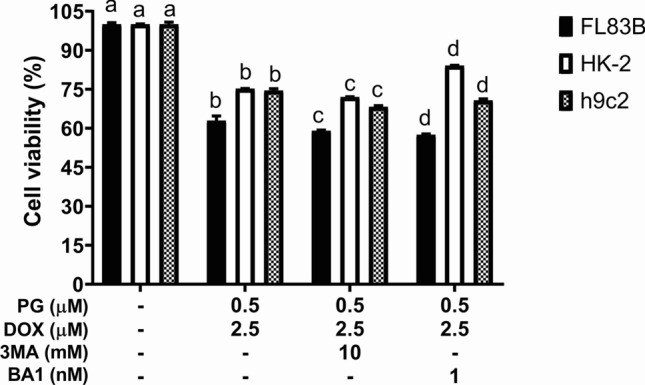
Cytotoxic alteration of PG/Dox by addition of autophagic inhibitors in normal cells. FL83B, HK-2, and h9c2 cells were treated with PG coupled with 3-methyladenine (3MA) or bafilomycin A1 (BA1) for 12 h following by Dox for 12 h, respectively. Results were normalized with untreated control and shown in mean ± SD from four independent experiments. “*” and “#” were represented to significantly different with untreated control and PG/Dox alone, respectively

**Fig. 3:**
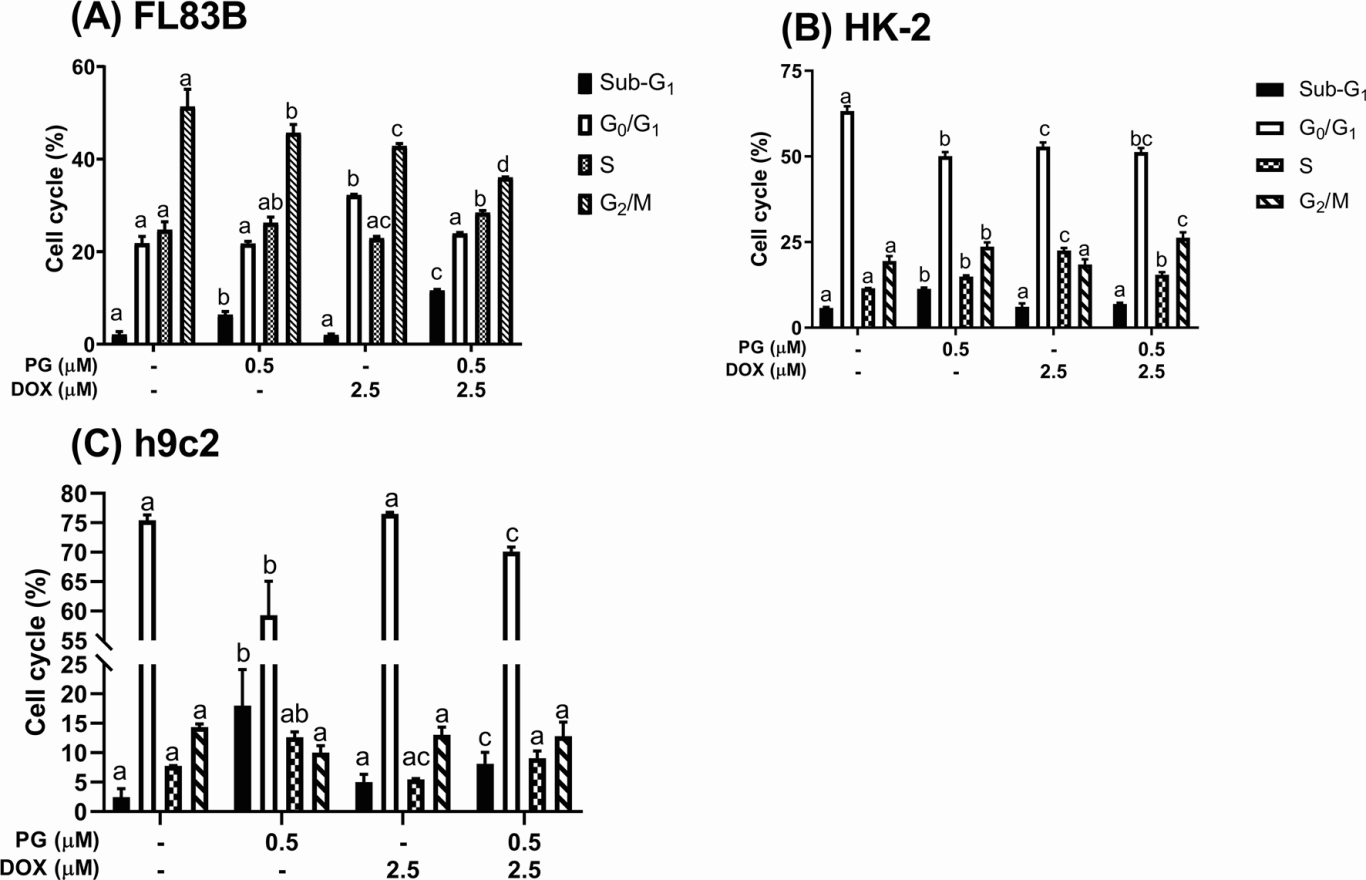
Cell cycle change in normal cells after PG/Dox treatment. FL83B, HK-2, and h9c2 were treated with PG and Dox, respectively, stained with PI, and determined intracellular fluorescent intensity by flow cytometry. Data were summarized from three independent experiments and shown in mean ± SD which were marked with “*” or “#” as significantly different (*p* < *0.05*) with untreated control or Dox alone

PG-priming reinforced Dox’s cytotoxicity via activating autophagy [19]. Therefore, autophagy might be stimulated in normal cells by PG-priming. Moreover, cell viability slightly decreased from combining with autophagic inhibitors except for HK-2 coupling with bafilomycin A1 (Fig. 2). This result revealed the different mechanisms of PG/Dox combination and the autophagy might play a protective role in normal cells instead of cytotoxic role in OSCC [19]**.** Afterward, cell cycle analysis was performed to evaluate the linkage of apoptosis and PG/Dox cytotoxic enhancement in normal cells. When compared with Dox as a baseline, sub-G_1_ in PG/Dox-treated FL83B and h9c2 cells was significantly raised, whereas HK-2 showed no significant differences (Fig. 3). In FL83B cells, elevated sub-G_1_ was contributed from decrease of G_0_/G_1_ and G_2_/M. In h9c2, increase of sub-G_1_ mainly came from G_0_/G_1_ decrease (Fig. 3). Moreover, S phase in PG/Dox-treated FL83B and h9c2 cells significantly elevated from 22.9 ± 0.4% to 28.4 ± 0.4% (FL83B) and 5.5 ± 0.1% to 9.1 ± 1.2% (h9c2), which was associated with the topoisomerase IIb inhibitory property of Dox (Fig. 3). In HK-2, it was interesting that S phase decreased and G_2_/M phase significantly increased in PG/Dox group when compared with Dox alone group (Fig. 3). That is, cytotoxic enhancement of PG-priming in HK-2 might be achieved through a different approach that has no linkage with autophagy. Importantly, all three cell lines showed sub-G_1_ increases in the PG alone group and were higher than the Dox-treated group, which meant that PG might be more toxic than Dox in normal cells. Detail triggers of PG-induced Dox cytotoxic enhancement were further examined via three strategies: oxidative stress, Dox influx, and Dox metabolism.

### Discover the triggering mechanism of PG/Dox synergism in normal cells

#### Dox influx alteration after PG-priming

**Fig. 4:**
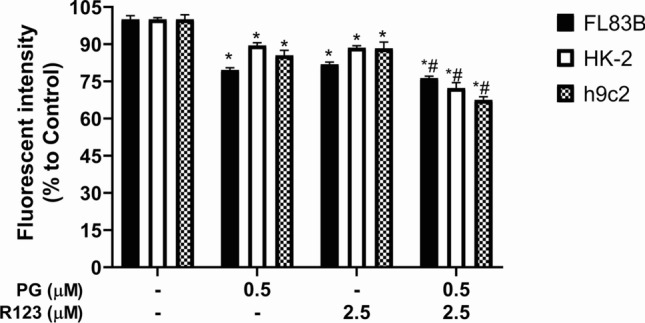
Normal cells’ Dox influx altered by PG-priming. Normal cells were treated with PG prior to rhodamine 123 (R123) and then intracellular fluorescent intensity was measured. Data were normalized to untreated control and shown in mean ± SD from four independent experiments. Significant difference (*p* < *0.05*) was labeled with “*” or “#” as compared with untreated control (*) or Dox alone (#)

In our previous study, PG-induced autophagy that increased Dox accumulation through upregulating veiled Dox importer expression has been demonstrated [19]**.** Accordingly, Dox influx would be the first strategy for testing the mechanism of PG-promoted Dox cytotoxicity in normal cells. In addition, only importer expressions were affected by PG-activated autophagy. Therefore, allosteric inhibition would not be determined during Dox influx analysis. Interestingly, Dox influx significantly declined in normal cells after 12-h PG-pretreatment (Fig. 4). This result excluded the involvement of Dox influx in PG-promoted Dox cytotoxicity.

#### Dox-related metabolism alteration after PG/Dox synergism

**Fig. 5:**
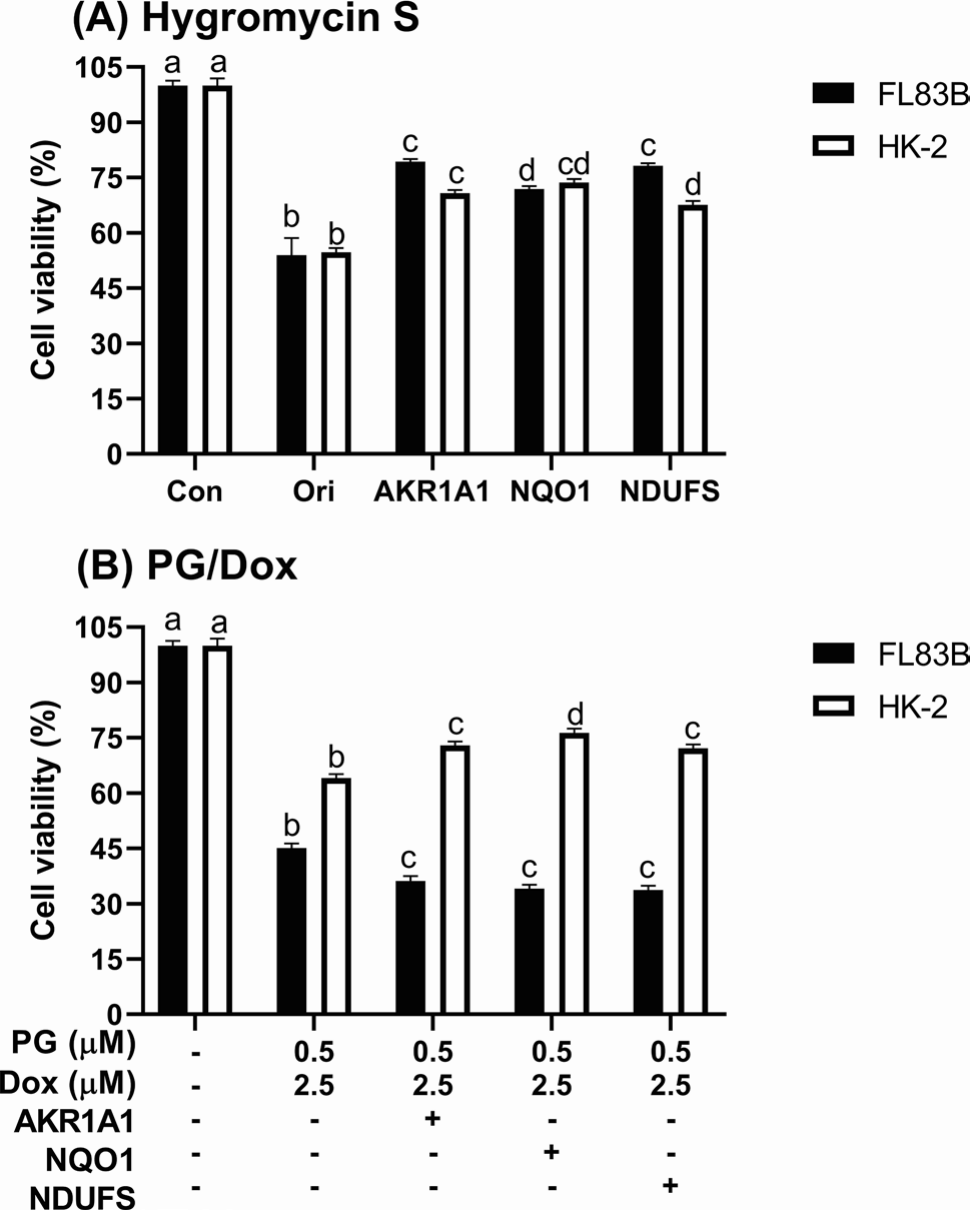
Cytotoxic change of PG/Dox synergism in normal cells after gene overexpression. Normal cells were overexpressed AKR1A1 and NQO1 via lentivirus transfection and selected through hygromycin S treatment. Then, transfected cells were treated with **a** hygromycin S and **b** PG/Dox to evaluate transfection success and impact of Dox-metabolic enzyme on PG/Dox synergism. Data were represented with mean ± SD from four independent experiments. Different letters on the columns meant significantly different to each other (*p* < *0.05*)

The reduction of Dox metabolism might contribute to Dox accumulation in either OSCC cells or normal cells. The experiment determined whether PG-priming altered Dox metabolism and subsequently raised Dox accumulation. The final enzymes of three metabolic pathways in Dox metabolism—Aldo/Keto reductase (AKR1A1), NAD(P)H quinone dehydrogenase (NQO1), and NADH:Ubiquinone oxidoreductase (NDUFS1, also known as mitochondrial complex 1)—were overexpressed by lentiviral activation particle sequentially selection with hygromycin S. In well-transfected cells, only HK-2 showed cell viability increases (Fig. 5). Combining results from gene overexpression, metabolic reduction of Dox was comprised in PG/Dox synergism in HK-2 cells, but not in FL83B cells.

### Sustained cytotoxicity of PG/Dox synergism in normal cells

**Fig. 6:**
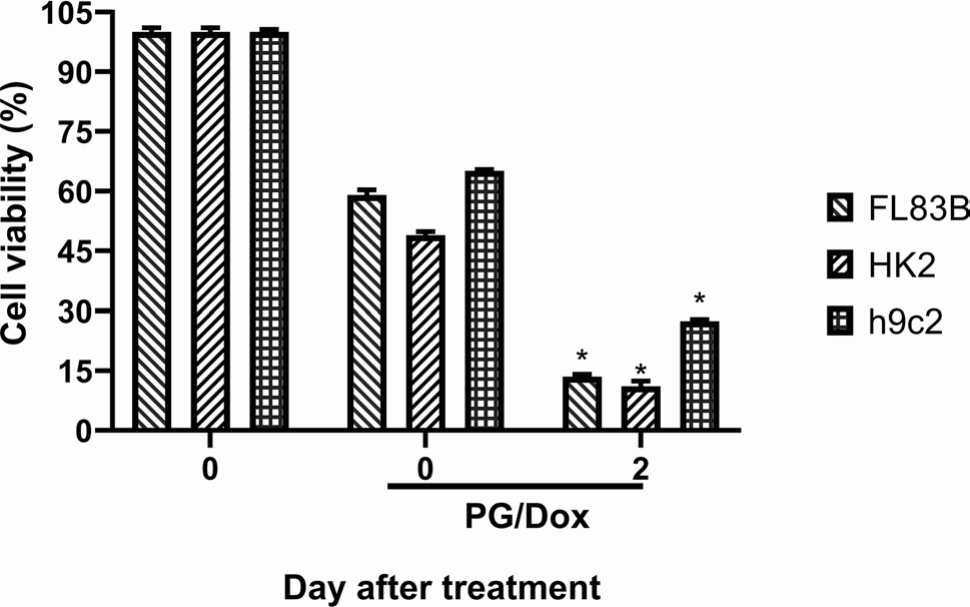
Sustained effect of PG/Dox in normal cells and OSCC. Normal cells were treated with PG/Dox for 12/12 h and renewed cultural medium for additional incubation for 2 days and determined cell viability by MTT assay. Results were shown in mean ± SD from 4 independent experiments and labeled with “*” as significantly different with PG/Dox treatment at Day 0

For clinical applications of PG/Dox synergism, the cytotoxic change after PG/Dox treatment in normal cells was measured. Subsequently, tested normal cells were treated with PG/Dox, renewed fresh cultural medium, and cultured additional for 2 days to determine post-treatment cytotoxic alteration in normal cells. Unfortunately, after 2 days culture, cell viability of all cells was continuously decreased to 13.5 ± 0.6% (FL83B), 11.1 ± 1.1% (HK-2), and 27.4 ± 0.4% (h9c2), respectively (Fig. 6), which meant sustained cytotoxic event was observed in normal cells and consequently could underestimate the cytotoxicity of PG/Dox synergism.

## Discussion

The result of this study can be seen as the two following approaches: (1) toxicity examination of PG/Dox synergism in normal cell; and (2) metabolic interference of Dox by PG/Dox synergism. This study demonstrated that cytotoxicity of PG/Dox synergism against normal cells was less than for OSCC cells and putatively might work through different pathways. Furthermore, PG/Dox synergism reduced Dox metabolism and led to increases in Dox accumulation in normal cells. However, sustained cytotoxicity of PG/Dox synergism could be found in normal cells and the under-estimation of cytotoxic efficacy against OSCC and its potential toxicity toward normal cells.

In the past 20 years, PG has been investigated in numerous biological functions with strong antitumor activity against a wide variety of cell type cancers [20]. Moreover, this study and our previous works have reported the chemo-sensitization of PG combined with Dox or paclitaxel for promoting the tumoricidal effect of these chemotherapeutic drugs [19, 21]. However, the pharmacodynamic (PD) and pharmacokinetic (PK) data for PG are still absent. The only information about PG’s PK/PD is its LD_50_, which is 26–30 μg/egg [22]. Chen et al*.* tested acute and hereditary toxicity of PG in mice, which showed the LD_50_ of PG in Kunming mice is larger than 10 g/kg and no putative hereditary toxicity was observed [23]. Other PK/PD information such as tissue distribution, absorption rate, eliminating route, and clearance is absent, which leads the clinical application of PG impossible. Accordingly, filling PK/PD data of PG is critical for clinical application of PG.

Due to high incidence in Dox-mediated cardiotoxicity, numerous researches work on the formulation or compound combination that could relief Dox-mediated toxicity. For ameliorating Dox-induced toxicity, antioxidants will be the first choice in filtrating candidates according to the ROS-inducing mechanism in Dox-induced toxicity and nature compounds with antioxidant activity being usually provided as a positive effect in relieving Dox-induced toxicity [9, 24]. The application of Traditional Chinese Medicine or herbal products in attenuation of Dox-induced cardiotoxicity, Qishen Yiqi Dropping Pills, beet root juice, and *Ginkgo biloba* extract shows different relieving mechanisms in vivo and in vitro in which Qishen Yiqi Dropping Pills upregulates VEGF expression in myocardio tissues, and beet root juice and *G. biloba* extract prevent the apoptosis of cardiomyocytes through modulating Bcl-2/Bax ratio [25–27]. Other than herbal products, 3,3′-Diindolylmethane and eriodictyol-7-O-glucoside activated Nrf-2-related anti-oxidant response element pathway to reduce Dox- and cisplatin-induced DNA damage [28, 29]. 6-Gingerol, Myricitrin, isorhamnetin, glabridin, and tanshinone IIA reduce cardiomyocyte apoptosis through activating growth factor signaling and preventing mitochondrial dysfunction [30–34]. Isodunnianol from *Illicium verum* activates protective autophagy through AMPK/ULK1 signaling pathway [35]. Comparing to our results, PG exacerbates Dox toxicity against oral squamous cell carcinoma cells through the augmenting of Dox influx which could not be observed in normal cell lines [19], which could not be found in identified Dox protectors. Our results elicit a new path for building distinct cytotoxicity of chemotherapy drug against cancer cell and normal cell to hinder its putative toxicity.

Another important finding of this study is the sustained cytotoxicity of PG/Dox synergism in normal cells even when the treatment came to an endpoint. From this finding evolved two concerned issues: (1) cytotoxicity of experimental observations needs extend to post-treatment; and (2) under-estimation of PG/Dox’s cytotoxicity. Mostly, sustained cytotoxicity is applied to describe the continuous release of packaged chemicals in a specific tissue such as wogonin nanoparticles against breast cancer [36], natamycin solid lipid nanoparticles in treating fungal keratitis [37], and self-assembly herbal hydrogels for neural inflammation [38]. For evaluations of small compound toxicity, this study is the first report on the impact of post-treatment cytotoxicity, while the detail mechanism after drug removal is entirely unclear. From previous study, oxidative stress was found to be one conceivable mechanism after drug removal which is the main cause of Dox-mediated cardiotoxicity and that also could be observed in normal cells during treatments [9]. When taking post-treatment cytotoxicity into considerations, current formulation of PG and Dox is appropriate and needs more investigation (dose or period of PG and Dox treated) to optimize the tumoricidal efficacy and minimize the normal cell toxicity.

## Conclusion

Of note, lowering normal cell cytotoxicity of PG/Dox synergism was reported and further reinforced the potential of PG/Dox for clinical application. Furthermore, the identification of PG in this study as a Dox-metabolic modulator resulted in the augment of Dox accumulation via either Dox importation or Dox metabolism. For future clinical application, the most critical events are the refilling PK/PD of PG and optimizing the PG/Dox regimen with considering cytotoxicity of post-treatment.
